# Deep Dive Into Familial Mediterranean Fever in a Child Without Fever

**DOI:** 10.7759/cureus.16968

**Published:** 2021-08-07

**Authors:** Vikash Jaiswal, Zouina Sarfraz, Trissa Paul, Furqan Ahmad Jarullah, Christine Zakhary

**Affiliations:** 1 Internal Medicine, AMA School of Medicine, Makati, PHL; 2 Internal Medicine, Fatima Jinnah Medical University, Lahore, PAK; 3 Internal Medicine, Avalon University School of Medicine, Ohio, USA; 4 Medicine, Jinnah Medical and Dental College, Karachi, PAK; 5 Internal Medicine, Ain Shams General Hospital, Cairo, EGY

**Keywords:** familial mediterranean fever, infection, colchicine, rare genetic diseases, autoinflammatory disease

## Abstract

This case report entails the details of a 12-year-old Egyptian boy who had recurrent episodes of shortness of breath, ascites, and pericardial effusions starting at the age of 10, returning with worsening symptoms in April of 2020. The lab findings indicated a critically elevated C-reactive protein (CRP) of 107.2 mg/L; a clinically notable inflammation process was festering. This case was all the more interesting as this boy did not present with a fever, making the diagnosis a difficult one. Nonetheless, genetic Mediterranean fever (MEFv) and polymerase chain reaction (PCR) testing confirmed the diagnosis of familial MEFv. Steroids and colchicine-salicylate decreased the frequency of the attacks and are now on half a dose of colchicine to keep his symptoms at bay. What we see here is the risk-to-benefit ratio of the therapeutic use of colchicine in children outweighs potential side effects such as nausea, vomiting, abdominal pain, diarrhea, kidney or liver failure. However, further research is needed to access better long-term treatment plans. Another key takeaway point that can be highlighted in this case is that the patient does not need to be febrile to diagnose FMF.

## Introduction

Familial Mediterranean fever (FMF) is an inherited autoinflammatory disorder characterized by recurrent episodes of fever, peritonitis, arthritis, pleuritis, and rash [1]. Some long-term complications of FMF include systemic amyloidosis and renal impairment [2]. FMF typically occurs in individuals of Mediterranean origin, but it may affect people of any ethnicity [3]. Patients usually present with inflammatory attacks before the age of 20, and symptoms persist for three days or less before they are spontaneously resolved [1,4]. The Mediterranean fever (MEFv) gene encodes for the Pyrin protein, which regulates inflammation and innate immunity [4]. The diagnosis of FMF is predominantly clinical and may be confirmed by MEFv gene mutation analysis [5]. Colchicine is the gold standard treatment of FMF that prevents recurrent seizures and is protective against the development of renal amyloidosis due to MEFv gene mutations [6]. In recent years, clinical management options of FMF have expanded, but data of Egyptian descent remain limited. In this report, an emphasis is placed on the clinical presentation and management of a 12-year-old male patient who had an afebrile presentation with MEFv gene mutation.

## Case presentation

A 12-year-old Egyptian male patient presented to the outpatient department with complaints of shortness of breath. On general physical examination, distant heart sounds and abdominal ascites were noted. A hemoglobin level of 10.6 g/dL indicating anemia was noted on laboratory testing, and he was admitted as an inpatient.

The echocardiogram revealed significant circumferential pericardial effusion, with no echocardiographic signs of cardiac tamponade. Thickening of the bilateral visceral and parietal pericardium was seen along with a shaggy appearing pericardial space and fibrinous threads all over, represented by maximal posterior dimensions measuring 1 cm and anterior dimensions measuring 0.5 cm in width. Other abnormalities on the echocardiogram were consistent with bilateral atrial enlargement, accompanied by a mild grade 1 diastolic dysfunction.

After these findings were noted, a pericardiocentesis was performed. The hematology report showed low hematocrit (HCT=35.2%), mean corpuscular hemoglobin (MCH=23.7 pg/cell), and elevated levels of red cell distribution width (RDW=15.7%). These levels were borderline normal.

A pleural fluid test was conducted to identify the cause of the pleural effusion. The pleural culture revealed a yellowish, turbid specimen with moderate amounts of pus on gram stain and lymphocytes on Leishman stain. The pericardial culture revealed a few pus cells on gram staining, however, the results were otherwise unremarkable. This patient’s sodium level was low (Na=136mmol/L) and the C-reactive protein (CRP=107.2 mg/L) was critically elevated consistent with severe inflammation.

The patient was put on a steroid treatment of prednisolone 20 mg, three times a day, on which improvement was noted and the patient was discharged. After he was tapered off of the steroid treatment he returned to the hospital six months post-initial presentation with similar symptoms, namely pericardial effusion and ascites.

The MEFv genetic mutation was positive, which is the gold standard test for FMF. A polymerase chain reaction (PCR) test was done to confirm the diagnosis of FMF, which returned positive as well. Colchicine 1 mg, an anti-inflammatory drug, was commenced daily to which the patient responded with dramatic improvement.

There was no presence of family FMF history; however, the response to colchicine, radiological and laboratory results aided in the workup at the onset. Because FMF mainly affects the people of Mediterranean and Middle Eastern origin, it is essential to note that the afebrile presentation was primarily supported by response to therapy and was corroborated by MEFv gene confirmation.

One year post initial presentation to the outpatient department, the patient was being administered with colchicine 0.5 mg daily, which kept his symptoms subsided. Echocardiogram and ultrasound tests were conducted once a month to monitor the patient's condition. On the last follow-up, the patient was progressing well, with no abnormal signs.

## Discussion

In addition to the 12-year-old boy’s case, we conducted a literature review of 23 FMF cases (Table 1). Of the 23 reported cases, 11 originated from Turkey, three from Italy, and one each from Armenia, Colombia, Germany, Iran, Israel, Japan, Morocco, and Pakistan. When noting the FMF criteria, gene mutations were noted in all the listed cases, with nine heterozygous MEFv gene mutations, six homozygous mutations, and seven otherwise unspecified MEFv gene mutations. The most commonly reported clinical manifestations of disease included fever, abdominal pain, bloody or mucous diarrhea, diffuse myalgia, and rashes. On documenting the family history, eight of the 23 studies confirmed a positive family history with a sibling, parent, or the first-degree relative diagnosed with FMF or being heterozygous/homozygous for the same mutation. The laboratory values suggested a rise in serum amyloid A (SAA) of the included cases, in addition to elevated CRP, ESR, ALT, and AST levels. The most common treatment option was colchicine, given its importance as the gold standard treatment. However, the inclusion of recent reports (2016-2021) led to the identification of cases with biologics and chemotherapeutic drugs being prescribed with documented recovery among patients.

**Table 1:** Characteristics of study findings of cases reported from 2016-2021. ALT=alanine transaminase; AST=aspartate aminotransferase; CRP=C-reactive protein; ESR=erythrocyte sedimentation rate; N/A=not available; RBBB=right bundle branch block; SAA=Serum amyloid A.

No	Author	Title	Country	Age	Gender	FMF criteria	Clinical Manifestations	Family History	Lab Values	Intervention	Outcome
1	Maggio et al.	Familial Mediterranean fever: an unusual cause of liver disease	Italy	10.6 years	Male	Homozygous MEFv gene mutation	Recurrent fever, aphthous stomatitis, rash, arthralgia, associated with abdominal pain, vomiting, lymphadenopathy	N/A	SAA=33 mg/L, CRP=24.8 mg/dL, ESR=86, AST and ALT were elevated	Colchicine	Recovery
2	Atmış et al.	Concomitance of familial Mediterranean fever and Gitelman syndrome in an adolescent	Turkey	9 years	Male	Homozygous MEFv gene mutation	Recurrent abdominal pain, fever, joint pain, and swelling for 3 years	The patient has one sibling and was diagnosed with FMF homozygous M694V	Hypokalemia, hyponatremia, hypomagnesemia	Colchicine	Recovery
3	Javascript et al.	Tofacitinib for familial Mediterranean fever: a new alternative therapy?	Colombia	16 years	Male	Heterozygous MEFv gene mutation	Recurrent fevers, cutaneous rash, and recurrent abdominal pain with diarrhea	N/A	N/A	Tofacitinib	Recovery
4	Yasuda et al.	Canakinumab Eliminates Resistant Familial Mediterranean Fever in a Japanese Girl	Japan	7 years	Girl	Heterozygous MEFv gene mutation	Recurrent febrile attacks including abdominal pain lasting 2–3 days	N/A	Elevated CRP	Canakinumab	Recovery
5	Yıldırım et al.	Protracted febrile myalgia as a challenging manifestation of familial Mediterranean fever: case-based review	Turkey	Median=6 years	3 Male; 2 Female	MEFv gene mutation	Severe myalgia, fever, abdominal pain, diarrhea, and arthralgia/arthritis	N/A	N/A	Corticosteroids, NSAIDs, anakinra, anti-interleukin-1	⅖ partial recovery, ⅗ shown full recovery to prednisolone while 2 have complete recovery with Anti IL-1 agent
6	Gökçe et al.	Polyarteritis nodosa in case of familial Mediterranean fever	Turkey	14 years	Male	Homozygous MEFv gene mutation	Fever, diffuse myalgia, abdominal pain, purpura	N/A	Leukocytosis, normal platelets, hemoglobin 13.3 normal urea and creatinine ESR 93 mm/h, CRP 193 mg/L, fibrinogen 6.68 mg/dL	Prednisolone, azathioprine, colchicine	Recovery
7	Frenkel et al.	A novel treatment of temporomandibular joint arthritis as a complication in familial Mediterranean fever-literature review and a case report	Israel	14 years	Female	Homozygous MEFv gene mutation	Painful swelling and redness over the involved TMJ area and severe trismus	Two siblings were diagnosed with FMF	High level of CRP (71) was noted	Colchicine	Recovery
8	Beyitler et al.	A case of familial Mediterranean fever having intermittent leukopenia	Turkey	13 years	Female	Heterozygous MEFv gene mutation	Recurrent pain, swelling, and hyperemia attacks on her ankle	Mother and uncle were diagnosed with FMF in past	Elevated ESR, CRP, SAA, leukopenia, and neutropenia	Colchicine	Not recovered fully
9	Yildirim et al.	Chronic non-bacterial osteomyelitis coexistent with familial Mediterranean fever	Turkey	11 and 13 years	Males	Heterozygous MEFv gene mutation	Case 1: Persistent swelling at medial region of right clavicle, relapsing fever and concomitant osteomyelitis. Case 2: Fever, swelling, warmth of left shoulder	N/A	Case 1: Elevated acute phase reactant, leukocytosis, anemia Case 2: Mild anemia, elevated acute-phase reactant	NSAIDs, colchicine, methotrexate	Recovery
10	Maggio et al.	Kawasaki disease triggered by EBV virus in a child with familial Mediterranean fever	Italy	3 years	Male	Heterozygous MEFv gene mutation	Fever, non-secretive conjunctivitis, lymphadenitis of the neck, generalized, fixed rash	A 6-year-old and the father had the same gene mutation	Leukocytosis, hyponatremia (128 mEq/L), hypoalbuminemia, elevated CRP, ESR, D-dimer, AST, ALT, SAA	Intravenous immune globulin, acetyl salicylate acid, h	Recovery
11	Farjadian et al.	A new MEFV gene mutation in an Iranian patient with familial Mediterranean fever	Iran	12 years	Male	MEFv gene mutation	Recurrent episodes of fever abdominal pain, nausea, vomiting, and general myalgia	N/A	Elevated CRP, ESR	Colchicine	Recovery
12	Baysal et al.	Transient pancytopenia and granulocyte abnormalities after suicide attempt with colchicine in a patient with familial Mediterranean fever	Turkey	16 years	Female	MEFv gene mutation	Vomiting, Diarrhea	N/A	Pancytopenia	Colchicine	Recovery
13	Cazzolla et al.	Orthopedic and orthodontic management in a patient with DiGeorge Syndrome and familial Mediterranean fever: a case report	Italy	8 years	Male	MEFv gene mutation	Recurrent fever episodes, arthralgias, polyserositis, hepatic-splenomegaly	Mother and maternal cousin of first degree were affected	N/A	Immunosuppressive agents, colchicine, antihypertensive therapy, calcitriol, erythropoietin	N/A
14	Gökçe et al.	Polyarteritis nodosa in case of familial Mediterranean fever	Turkey	14 years	Male	Homozygous MEFv gene mutation	Fever, diffuse myalgia, abdominal pain and purpura	No family history	Leukocytosis, elevated ESR, CRP, and proteinuria	Colchicine	Recovery after a long time
15	Demir et al.	Systemic amyloidosis in a patient with familial Mediterranean fever and Hodgkin lymphoma: a case report	Turkey	12 years	Female	Heterozygous MEFv gene mutation	Abdominal pain and episodes of fever	Consanguinity marriage; three sons of her uncle had been diagnosed with FMF	Elevated SAA, CRP, and ESR	Colchicine (2 mg/day); anakinra (2 mg/kg/day); doxorubicin, bleomycin, vinblastine, and dacarbazine (ABVD)	Recovery
16	Aydoğdu et al.	An extraordinary complication in a child with combined familial Mediterranean fever and inflammatory bowel disease: multiple ileal perforations	Turkey	5 years	Female	Heterozygous MEFv gene mutation	Fever, abdominal pain, vomiting	N/A	Elevated CRP and ESR	Colchicine, oral steroids, canakinumab, anakinra, infliximab	Recovery
17	Sag et al.	Neonatal ulcerative colitis associated with familial Mediterranean fever: a case report	Germany	3 months	Female	Homozygous MEFv gene mutation	Bloody and mucous diarrhea, two episodes of high fever	N/A	Elevated CRP	Colchicine	Recovery
18	Ceylan et al.	Intermittent right bundle branch block in a child with familial Mediterranean fever	Turkey	8 years	Male	Heterozygous MEFv gene mutation	N/A	N/A	Elevated Inflammatory biomarkers. RBBB could be associated with FMF or Colchicine	Colchicine	Symptom-free except for incidental finding of RBBB
19	Zerkaoui et al.	A novel single variant in the MEFV gene causing Mediterranean fever and Behçet’s disease: a case report	Morocco	10 years	Female	MEFv gene mutation	Periodic fever, abdominal pain, mucocutaneous symptoms, and joint pain	The mother was normal, and her father was heterozygous for the same mutation	Elevated CRP	Colchicine, methylprednisolone, opioids, intravenous anticoagulation for sinus thrombosis	Recovery
20	Yoldas et al.	Massive pericardial effusion and tamponade can be a first sign of familial Mediterranean fever	Turkey	10 and 13 years	Male, Female	Heterozygous MEFv gene mutation	Chest pain, dyspnea, and fever	N/A	Elevated ESR, CRP	Colchicine	Recovery
21	Shahsuvaryan et al.	Is plasmapheresis a potential treatment for familial Mediterranean fever patients resistant or intolerant to colchicine?	Armenia	17 years	Female	MEFv gene mutation	Recurrent attacks of fever and abdominal pain	N/A	Elevated ESR and CRP	Colchicine	N/A
22	Maggio et al.	PAPA and FMF in two siblings: possible amplification of clinical presentation? A case report	Italy	8.4 and 16 years	Male	Homozygous MEFv gene mutation	Fever, oral aphthous stomatitis, abdominal pain, arthritis, undefined dermatitis, vomiting, diarrhea	Both parents were heterozygous for the same mutation p.M680I	Elevated AST, ALT, CRP, ESR, SAA, leukocytosis with neutrophilia	Colchicine	Recovery
23	Hong et al.	Autoinflammation due to homozygous S208 MEFV mutation	Pakistan	9 and 12 years	Male	MEFv gene mutation	Recurrent fever, oral ulceration, purpuric rashes, arthralgia, eosinophilia, and osteitis	No family history of genetic testing	Elevated SAA, CRP, and leukocytosis	Lipopolysaccharide, IL-1 blockade	N/A

The striking feature of our case presentation is the fact that the young male patient did not present with fever, similar to findings observed by Hotta and Mattiassich et al. [7,8]. Cekin and Aslan et al. [9,10] had identical complaints to our patient comprising abdominal ascites. Aslan et al. [10], however, did not note abdominal pain, which was identified by Tatar and Lee et al. [11,12], but without distention secondary to ascites. Incidentally, it was discovered soon after the initial workup that our patient also had added heart sounds, who was later diagnosed to have pericardial effusion. Abukhalaf and Ceylan et al. [13,14] also reported cardiac complications among their patients, namely cardiac amyloidosis and right bundle branch block, respectively.

Despite the differences in age groups, clinical presentations, family history, or laboratory results, colchicine remains the first-line treatment modality for a majority of the tabulated patients, with documented recovery. However, Ceylan et al. point out an unprecedented view of FMF, where colchicine may exacerbate pre-existing cardiac conditions [14]. Even though the presentation was an isolated incident, it is pivotal to monitor colchicine during and post-administration on a regular basis. When identifying clinical practice points highlighted with our case and subsequent tabulation of recent trends in treatment, it is pertinent to note the European League Against Rheumatism (EULAR) recommendations for FMF management supported by the best evidence available [15]. As with EULAR recommendations, the best management strategy for FMF is to control acute attacks, minimize any chronic subclinical inflammation, improve the acceptable quality of life, and prevent recurring complications.

While FMF is mostly a clinical diagnosis, laboratory analysis may reveal elevated white blood cell count with peaked neutrophil. An elevation of acute-phase reactants, such as erythrocyte sedimentation rate (ESR), SAA, and CRP, is not uncommon. Radiological testing may be utilized to reveal other causes of abdominal pain, such as the acute abdomen. Gene mutation testing may be utilized to confirm the diagnosis with atypical presentations, particularly in non-endemic areas.

Overall, a central challenge for interprofessional healthcare teams managing FMF is to reach a diagnostic conclusion. Education about the etiology, family history, and testing options, in addition to associating typical or atypical clinical presentations to FMF, is necessary. Once the diagnosis is established, the clinician, nurse, and other providers ought to work closely. The use of colchicine, in addition to biologics and chemotherapeutic drugs along with noting the normal or abnormal clinical presentation, is essential in any clinical setting.

## Conclusions

FMF is the most common autoinflammatory disease. The usual presentation of FMF in patients is fever; but in this case, the patient was afebrile whereas the diagnosis was confirmed on PCR testing. Colchicine, which has been the prescribed treatment for FMF since 1972, has shown a promising impact on patient conditions and is currently the first-line treatment for management. Further research is needed to access better long-term treatment plans such as the use of biologics namely anti-interleukin 1, anti-interleukin 6, Janus kinase inhibitors, and anti-TNF drugs. It is recommended that colchicine-resistant and intolerant cases be monitored for certain cytokines, along with genetic studies to improve clinical outcomes and compliance to treatment in endemic countries.

## References

[1] Simon A, van der Meer JW, Drenth JP (2005). Familial Mediterranean fever--a not so unusual cause of abdominal pain. Best Pract Res Clin Gastroenterol.

[2] Kastner DL (1998). Familial Mediterranean fever: the genetics of inflammation. Hosp Pract (1995).

[3] Ben-Chetrit E, Touitou I (2009). Familial mediterranean fever in the world. Arthritis Rheum.

[4] Mansour AR, El-Shayeb A, El Habachi N (2019). Molecular patterns of MEFV gene mutations in Egyptian patients with familial Mediterranean fever: a retrospective cohort study. Int J Inflam.

[5] Bonyadi M, Esmaeili M, Karimi A, Dastgiri S (2010). Common Mediterranean fever gene mutations in the Azeri Turkish population of Iran. Genet Test Mol Biomarkers.

[6] Fietta P (2004). Autoinflammatory diseases: the hereditary periodic fever syndromes. Acta Biomed.

[7] Hotta Y, Kawasaki T, Kotani T (2020). Familial mediterranean fever without fever. Intern Med.

[8] Mattiassich G, Semlitsch G, Nadler K, Rainer F (2013). Familial Mediterranean fever without fever as a cause of monoarthritis. BMJ Case Rep.

[9] Cekin AH, Dalbudak N, Künefeci G (2003). Familial mediterranean fever with massive recurrent ascites: a case report. Turk J Gastroenterol.

[10] Aslan M, Demir G, Esen R (2012). A rare cause of massive ascites: familial Mediterranean fever. Turk J Gastroenterol.

[11] Tatar E, Uslu A, Simsek C, Aykas A, Bozkaya G, Imamoglu C (2017). Late diagnosis of E148Q mutation-positive familial mediterranean fever in a kidney transplant patient with fever of unknown origin: a case report. Exp Clin Transplant.

[12] Lee CG, Lim YJ, Kang HW (2014). A case of recurrent abdominal pain with fever and urticarial eruption. Korean J Gastroenterol.

[13] Abukhalaf SA, Dandis BW, Za'tari T, Amro AM, Alzughayyar TZ, Rajabi YA (2020). Familial mediterranean fever complicated by a triad of adrenal crisis: amyloid goiter and cardiac amyloidosis. Case Rep Rheumatol.

[14] Ceylan Ö, Ceylan Y (2018). Intermittent right bundle branch block in a child with familial mediterranean fever. Cardiol J.

[15] El Hasbani G, Jawad A, Uthman I (2019). Update on the management of colchicine resistant familial Mediterranean fever (FMF). Orphanet J Rare Dis.

